# Decompressive Abdominal Laparotomy for Abdominal Compartment Syndrome in an Unengrafted Bone Marrow Recipient with Septic Shock

**DOI:** 10.1155/2010/102910

**Published:** 2010-06-23

**Authors:** Derrick J. N. Dauplaise, Sean J. Barnett, Jason S. Frischer, Hector R. Wong

**Affiliations:** ^1^Division of Critical Care Medicine, Department of Pediatrics, Cincinnati Children's Hospital Medical Center, 3333 Burnet Avenue MLC 2005, Cincinnati, OH 45229, USA; ^2^Department of Surgery, Cincinnati Children's Hospital Medical Center, 3333 Burnet Avenue MLC 2005, Cincinnati, OH 45229, USA

## Abstract

*Objective*. To describe a profoundly immunocompromised (panleukopenia) child with septic shock who developed abdominal compartment syndrome (ACS) and was successfully treated with surgical decompression. *Design*. Individual case report. *Setting*. Pediatric intensive care unit of a tertiary children's hospital. *Patient*. A 32-month-old male with Fanconi anemia who underwent bone marrow transplantation (BMT) 5 days prior to developing septic shock secondary to *Streptococcus viridans* and *Escherichia coli* ACS developed after massive fluid resuscitation, leading to cardiopulmonary instability. *Interventions*. Emergent surgical bedside laparotomy and silo placement. *Measurements and Main Results*. The patient's cardiopulmonary status stabilized after decompressive laparotomy. The abdomen was closed and the patient survived to hospital discharge without cardiac, respiratory, or renal dysfunction. *Conclusions*. The use of laparotomy and silo placement in an unengrafted BMT patient with ACS and septic shock did not result in additional complications. Surgical intervention for ACS is a reasonable option for high risk, profoundly immunocompromised patients.

## 1. Introduction

Intra-abdominal hypertension (IAH) leading to abdominal compartment syndrome (ACS) is increasingly being recognized as a cause of morbidity and organ dysfunction, which includes respiratory insufficiency, compromised renal perfusion, and decreased cardiac output from impaired venous return, but can included any organ dysfunction caused by IAH. Clinical intra-abdominal pressure measurements and surgical decompression with silo placement are well described and are becoming the mainstays of treatment [1, 2]. The reported incidence of ACS in critically ill children has been low (0.6%–4.7%); however, mortality in the setting of ACS has been high (50%–60%) even after intervention with surgical decompression of the abdomen [3–5]. Complications of surgical abdominal decompression for ACS include transient hypotension, bleeding, visceral damage, and surgical site infections [1, 6, 7]. Inability to achieve primary closure is associated with increased risk of ventilator-associated pneumonia (VAP), blood stream infection (BSI), and surgical site infection (SSI) [7]. One might anticipate that the risk of these complications would be substantially higher in a chronically ill patient with profound immunosuppression, such as a patient in the early period post bone marrow transplantation (BMT) with panleukopenia. Accordingly, the potential risk to benefit ratio of laparotomy for ACS in patients with significant comorbidities needs to be carefully considered. Currently, there are no reports of surgical decompression for ACS in a pediatric or adult BMT patient with septic shock.

## 2. Case

A 2 1/2-year-old male (10.9 kg), with a history of Fanconi anemia presented to the PICU for treatment of septic shock. His past medical history was significant for a recent (5 days earlier) BMT with a matched unrelated donor. His past medical history also included Fanconi anemia-related musculoskeletal involvement, intrauterine growth retardation, dilated cerebral ventricles, colpocephaly, plagiocephaly, hearing loss, and pelvic kidney. He previously underwent surgical correction of a VSD, PDA, a small ASD, and coarctation of the aorta at 10 months of age and sustained paralysis of the left vocal cord resulting in aspiration and poor feeding. There were no significant residual cardiac lesions at the time of presentation to the PICU. Preparative bone marrow ablation regimen included antithymocyte globulin, Cytoxan, and Fludarabine. 

He developed polymicrobial septic shock secondary to *Streptococcus viridans* and *Escherichia coli,* 5 days after BMT. At the time of presentation to the PICU, his peripheral total white blood cell count was 0.1 K/mcL, with a hemoglobin of 9.7 gm/dL, and a platelet count of 4 K/mcL. Antibiotics initially were gentamicin, piperacillin/tazobactam, and vancomycin; however, after admission to the PICU, treatment was changed to meropenem, tobramycin, and vancomycin. Due to profound shock and profound capillary leak he required massive fluid resuscitation: 70 ml/kg in the first 60 minutes and 330 ml/kg in the first 24 hours including normal saline, albumin, red blood cells, fresh frozen plasma, and platelets. Inotrope and vassopressor support peaked at time of decompression with an epinephrine infusion of 0.6 mcg/kg/min and vasopressin at 4 milliunits/kg/hr. Increasing iontropy and vassopressor support would increase blood pressure temporally; however, an increase in tachycardia and hypotension would quickly return. Ventilatory support was initiated as pressure-control pressure-support with a peak inspiratory pressure (PIP) of 18 cm H_2_O and positive end expiratory pressure (PEEP) of 5 cm H_2_O, resulting in an oxygenation index (OI) of less than 2. His mechanical ventilation requirements rapidly increased in parallel to the capillary leak and the development of ACS (Table 1). 

The patient developed multiple episodes of bradycardia and hypotension 20 hours after arriving in the PICU, requiring cardiopulmonary resuscitation, and a persistent lactic acidosis in the range of 6 mmol/L. Serial exams of the abdomen during this time revealed an increasingly large and tense abdomen. Serial bladder measurements were consistent with the clinical findings in that bladder pressures which quickly increased from 20 cm H_2_O to 43 cm H_2_O. Temporally related to these findings were the development of oliguria and progressively worsening oxygenation/ventilation. 

Based on the abdominal exam, the serial bladder pressure measurements consistent with IAH, oliguria, and the severe cardiopulmonary instability, a diagnosis of ACS was made. Medical management was initiated with improving the abdominal wall compliance with sedation, neuromuscular blockade, and keeping the head of the bed no greater then 30 degrees, evacuation of intraluminal contents with nasogastric decompression, and organ support with optimizing abdominal perfusion pressure with iontropy and oxygenation with alveolar recruitment. The continued requirement for fluid resuscitation and escalating iontropic support did not allow for the use of diuretics. Despite these measures, bladder pressures increased and discussions ensued regarding surgical decompression of the abdomen. The profound capillary leak and aggressive fluid resuscitation were the primary components in developing ACS in this patient. The primary concern with proceeding to surgical decompression was the clinical perception that the patient would be at high risk for surgical and/or infectious complications given his severely immuncompromised status and concern for his overall prognosis. Otherwise, the patient was considered to be a good candidate for surgical decompression because, (1) end-organ failure had not yet progressed to the point of irreversibility, (2) the primary cause of IAH/ACS (i.e. sepsis) was potentially treatable, (3) it was expected that there would be eventual successful engraftment of the BMT, and (4) it appeared that the patient would die from refractory shock within the next 24 hours if the ACS was not directly addressed. The surgical team decided on an open laparotomy to accommodate the possibility of oganomegaly from the edema, as percutaneous drainage may not have alleviated enough pressure. The decision was made to proceed urgently with surgical decompression of the abdomen after discussion with the family, BMT, and surgical team.

Bedside decompressive laparotomy was performed with placement of a silo. The surgical findings were significant for voluminous clear ascites under tension and grossly edematous, but normal appearing abdominal viscera. The edematous viscera readily extravasated into the silo. Thirty minutes after decompression, the vasopressin infusion was discontinued and the epinephrine infusion was decreased to 0.5 mcg/kg/min, urine output increased to 1 ml/kg/min, and the lactic acid was less then 4 mmol/L. Four hours after the decompression, HFOV was converted to conventional ventilation with an OI of 9.5 (Table 1). Urine output remained at 1.5 ml/kg/hr despite a trial of diuretics with furosemide and serum creatinine peaked at 1.1 mg/dL from 0.4 mg/dL at presentation. Because the patient had substantial anasarca, continuous venovenous hemofiltration (CVVH) was initiated two days after decompression. The primary indication for CVVH was more efficient fluid removal in order to close the abdomen in a timely manner. The silo was removed and the abdomen was closed 5 days after initial bedside decompression in the operating room. He completed 6 weeks of antibiotics until his bone marrow transplant had engrafted. CVVH was continued for 6 more days until renal function improved to the point of only requiring diuretic therapy. He made a full recovery and is alive and well 5 months later.

## 3. Discussion

Compartment syndrome occurs when pressure in any closed anatomical space exceeds the perfusion pressure, leading to tissue ischemia from compromised blood flow. In a similar manner to that of extremities following orthopedic trauma, the abdominal cavity is at risk for elevated intra-abdominal pressure (IAP) due to bowel/tissue edema and fluid collecting in the abdominal cavity. Intra-abdominal hypertension (IAH) specifically refers to elevated pressure in the abdominal cavity; however, when there is organ failure that occurs due to this increased IAP, this is referred to as abdominal compartment syndrome (ACS). ACS is not based solely on pressure measurements, but rather on the pathophysisological compromise that results from the increasing IAP [8–10]. ACS can lead to increased intracranial pressure, abdominal organ ischemia, and thrombosis from venous stasis leading to multisystem organ failure and death [11–14]. There is now an ACS organization to assist in standardizing definitions and treatment guidelines (http://www.abdominal-compartment-syndrome.org/ and http://www.wsacs.org/).

The majority of early ACS literature is in surgical trauma patients, but this syndrome is not confined to trauma patients. ACS is observed in medical and surgical patients, even with low intra-abdominal pressures [3]. The highest incidence of IAH reported in the literature is in medical patients, especially those with severe sepsis [15–18]. Patients that require aggressive volume resuscitation and vasopressor support are at increased risk for developing bowel edema and IAH leading to renal, pulmonary, and cardiovascular dysfunction and ACS [19]. Predisposing factors for patients with abdominal injuries leading to ACS include extensive abdominal injury, massive transfusions, abdominal contamination, inadequate resuscitation leading to gut mucosal acidosis and bowel edema, coagulopathy, and closure of fascia under tension [11]. Furthermore, those who have encountered direct peritoneal injury from trauma, surgery, or localized inflammation (such as bowel obstruction, colitis, pancreatitis, necrotizing enterocolitis, mesenteric ischemia, Wilms tumor, neuroblastoma, and gastroschisis) are also at risk [3, 19–23]. 

Compared to the adult literature, relatively less research has been published regarding ACS in critically ill children. Beck et al. suggested that ACS is less frequent in children than adults, occurring in less than 1% of pediatric ICU admissions with an associated 60% mortality rate [3]. In contrast, Ejike and Mathur. reported an ACS incidence of 17.6% in mechanically ventilated children, which is higher than the occurrence in adults [24]. With improvements in critical care, more critically ill patients are surviving the initial resuscitation and IAP monitoring should be considered in these patients. The clinical exam alone has shown to be unreliable, and it is recommend to use IAP monitoring in conjunction with the clinical exam [25, 26]. 

Critically ill children should have intra-abdominal pressures measured when rapid abdominal distension develops, especially in the setting of organ dysfunction. The gold standard to measure intra-abdominal pressure is a peritoneal catheter, which is invasive [27, 28]. Other less invasive options include pressure transduction through placement of a tube in the stomach, bladder, or rectum. Measurement of IAP can also be determined with measurement of bladder pressures through a Foley catheter by using fluid inserted into the bladder to passively transmit intra-abdominal pressure to the monitor. Appropriate volumes must be used to avoid overdistension of the bladder, because as the bladder wall begins to stretch pressure, measurements will rise to reflect bladder wall compliance, rather than intra-abdominal pressure. Studies suggest that either use of 1 ml/kg intravesically (not exceeding 20–25 ml) or just using 6 ml infusion volume in pediatric patients is needed to obtain accurate intra-abdominal pressure [29, 30]. Various ranges have been reported that may require decompression of the abdomen from 15 to 25 mm Hg (20 to 34 cm H2O) [11, 12, 31, 32]. Alternatively, it has been proposed that it is more appropriate to use adverse physiological consequences of increased intra-abdominal pressure alone as criteria for decompression, such as progressive renal or cardiopulmonary insufficiency [33]. Specific pressure cutoffs are not known and many experts recommend that the entire clinical picture must be taken into account [11, 34]. 

Clinical recognition of cardiopulmonary complications secondary to IAH leading to ACS has increased since the 1960s in patients with ascites and was addressed at this time by paracentesis [35–38]. Medical management of IAH consists of many levels of interventions including removal intralumenal contents such as by nasogastric tube; improving abdominal wall compliance with positioning or neuromuscular blockade; optimizing fluid administration with careful resuscitation, diuresis, or use of hemodialysis; and optimization of perfusion globally and locally. Medical management improves survival and prevents progression to ACS [8, 10, 39–41]. Progression from IAH to ACS changes the patient's status from an urgent medical process to a surgical emergency because end stage tissue ischemia is present creating more inflammation [42]. There is universal agreement that the only treatment for ACS is abdominal decompression [1, 5, 8–11, 13, 32, 42].

## 4. Summary

From review of the literature and to our knowledge this is the first case report of decompressive laparotomy for abdominal compartment syndrome for a BMT patient with septic shock. This patient had several chronic issues related to his Fanconi anemia; however, his state of health was different from many BMT patients frequently encountered with sepsis in the ICU. We recognized that he was only 5 days out from his stem cell transplant and did not have an extensive history of medical treatments affecting other organ systems that would have placed him at a much higher risk. For example, it is established that pediatric BMT patients with primary respiratory failure do very poorly [43, 44]. The purpose of this case report is to describe a patient with high-risk factors that benefited from an intervention that may have been withheld because of his severely compromised immune status and high risk for surgical complications after decompressive laparotomy. Treatment of ACS requires clinical suspicion in patients who have risk factors such as abdominal trauma, acute pancreatitis, intra-abdominal abscess, intra-abdominal organ transplantation, closure of large ventral hernias, and capillary leak from inflammation following large volume resuscitation. Immediate decompression is required for patients who develop acute complications from ACS, such as pulmonary, cardiac, and renal compromise. Bladder pressure measurement can be helpful in patients without organ dysfunction with acute abdominal distension.

## Figures and Tables

**Table 1:** Ventilation parameters before and after abdominal decompression.

	Initial Settings	Prior to HFOV	At time Of Decompression	2 Hrs Post Decompression	4 Hrs Post Decompression
PIP cmH_2_O	18	38			30
PEEP cmH_2_O	5	12			10
Rate	20	30			26
MAP cmH_2_O	8	31			17
FiO2	0.5	1.0	1.0	0.6	0.6
Amplitude			55	30	
MAP cmH_2_O			32	24	
Hertz			8	8	
p_a_O_2_ mmHg	275	66	85	145	107
pCO_2_ mmHg	41	56	40	30	33
pH	7.27	7.31	7.4	7.56	7.46
OI	1.5	47	37.5	10	9.5
Lactate mmol/L	2.0	2.4	6.1	3.8	1.3

HVOF (high frequency oscillatory ventilation), PIP (peak inspiratory pressure), PEEP (positive end expiratory pressure), MAP (mean airway pressure), OI (oxygenation index).
